# Clinical *rel* mutations in *Staphylococcus aureus* prime pathogen expansion under nutrient stress

**DOI:** 10.1128/msphere.00249-23

**Published:** 2023-09-26

**Authors:** Edwin Chen, Marla G. Shaffer, Robert E. Bilodeau, Raymond E. West, Patrick J. Oberly, Thomas D. Nolin, Matthew J. Culyba

**Affiliations:** 1 Division of Infectious Diseases, Department of Medicine, University of Pittsburgh School of Medicine, Pittsburgh, Pennsylvania, USA; 2 Small Molecule Biomarker Core, University of Pittsburgh School of Pharmacy, Pittsburgh, Pennsylvania, USA; 3 Center for Evolutionary Biology and Medicine, University of Pittsburgh School of Medicine, Pittsburgh, Pennsylvania, USA; University of Nebraska Medical Center, Omaha, Nebraska, USA

**Keywords:** *Staphylococcus aureus*, bacteremia, stringent response, persistence, mrsa

## Abstract

Persistent infection by *Staphylococcus aureus* has been linked to the bacterial stringent response (SR), a conserved stress response pathway regulated by the Rel protein. Rel synthesizes (p)ppGpp “alarmones” in response to amino acid starvation, which enables adaptation to stress by modulating bacterial growth and virulence. We previously identified five novel protein-altering mutations in *rel* that arose in patients with persistent methicillin-resistant *S. aureus* bacteremia. The mutations mapped to both the enzymatic and regulatory protein domains of Rel. Here, we set out to characterize the phenotype of these mutations to understand how they may have been selected *in vivo*. After introducing each mutation into *S. aureus* strain JE2, we analyzed growth, fitness, and antibiotic profiles. Despite being located in different protein domains, we found that all of the mutations converged on the same phenotype. Each shortened the time of lag phase growth and imparted a fitness advantage in nutritionally depleted conditions. Through quantification of intracellular (p)ppGpp, we link this phenotype to increased SR activation, specifically during the stationary phase of growth. In contrast to two previously identified clinical *rel* mutations, we find that our *rel* mutations do not cause antibiotic tolerance. Instead, our findings suggest that *in vivo* selection was due to an augmented SR that primes cells for growth in nutrient-poor conditions, which may be a strategy for evading host-imposed nutritional immunity.

**Importance:**

Host and pathogen compete for available nutrition during infection. For bacteria, the stringent response (SR) regulator Rel responds to amino acid deprivation by signaling the cell to modulate its growth rate, metabolism, and virulence. In this report, we characterize five *rel* mutations that arose during cases of persistent methicillin-resistant *Staphylococcus aureus* bacteremia. We find that all of the mutations augmented SR signaling specifically under nutrient-poor conditions, enabling the cell to more readily grow and survive. Our findings reveal a strategy used by bacterial pathogens to evade the nutritional immunity imposed by host tissues during infection.

## INTRODUCTION

The bacterial stringent response (SR) is a conserved stress response pathway that is regulated by proteins of the RelA-SpoT Homologue (RSH) superfamily. RSH proteins synthesize and/or hydrolyze guanosine 5′-diphosphate 3′-diphosphate (ppGpp) and guanosine 5′-triphosphate 3′-diphosphate (pppGpp). ppGpp and pppGpp are often referred to collectively as (p)ppGpp, or alarmones, due to their role as second messenger molecules that affect bacterial physiology. Synthesis is accomplished by catalyzing the transfer of a pyrophosphate from adenosine 5′-triphosphate (ATP) to the 3′-hydroxyl of either guanosine 5′-diphosphate (GDP) or guanosine 5′-triphosphate (GTP). Hydrolysis of (p)ppGpp yields inorganic pyrophosphate and either GDP or GTP. (p)ppGpp acts both directly and indirectly to modulate various downstream processes, including DNA replication, transcription, translation, energy metabolism, and virulence (1, 2). Regulation of intracellular (p)ppGpp concentrations by RSH proteins enables the cell to adapt its growth rate, metabolism, and virulence expression to current environmental conditions.

**FIG 1:**
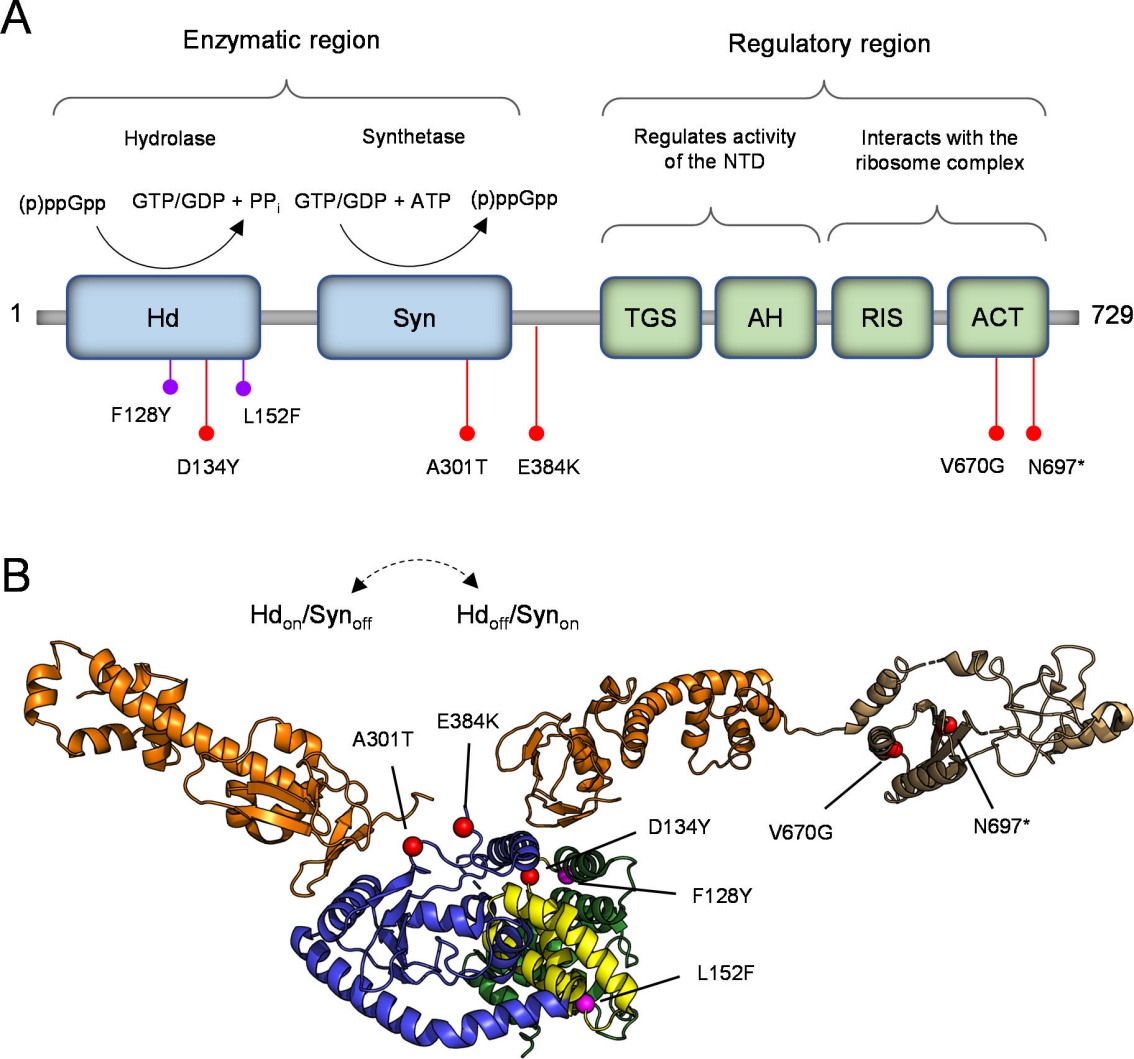
Rel domain architecture and location of clinical mutations. (**A**) The N-terminal enzymatic region containing the Hd and Syn domains is shown in blue. The C-terminal regulatory region containing the TGS, AH, RIS, and ACT domains is shown in green. The Hd domain catalyzes the hydrolysis of (p)ppGpp to yield inorganic pyrophosphate (PP_i_) and either GTP or GDP. The Syn domain catalyzes the transfer of pyrophosphate from ATP to either GTP or GDP to form (p)ppGpp. The mutations examined in this study are denoted with red markers. Previously identified mutations are denoted with purple markers. (**B**) Clinical Rel mutations mapped onto the structure of a Hd_on_/Syn_off_ RelΔRIS-CT from *B. subtilis* (PDB 6YXA) with the CTD of a Hd_off_/Syn_on_ Rel from *E. coli* (PDB 5KPV) superimposed. Domain colorings: hydrolase, green; central 3-helix bundle, yellow; synthetase, blue; TGS-AH, orange; RIS-ACT, tan. Rel mutations focused on in this study are denoted by red spheres. Previously identified mutations are denoted by purple spheres.

Most bacteria encode at least one “long” bifunctional RSH protein, Rel, which comprises an enzymatic N-terminal domain (NTD) and several regulatory C-terminal domains (Fig. 1A). The NTD contains distinct hydrolase (Hd) and synthetase (Syn) subdomains responsible for the hydrolysis and synthesis of (p)ppGpp, respectively. Four C-terminal domains [TGS (ThrRS, GTPase, SpoT), AH (Alpha-Helical), RIS (Ribosome Inter Subunit), ACT (Aspartate kinase, Chorismate mutase, TyrA)] are involved in binding the ribosome and regulating the reciprocal enzymatic activities of the NTD. Structural evidence suggests that Rel exists in two distinct conformational states to avoid futile substrate cycling: Hd_on_/Syn_off_ and Hd_off_/Syn_on_ (Fig. 1B) (3). Under nutrient-rich conditions, Rel is usually in a net hydrolysis state (Hd_on_/Syn_off_) to maintain low levels of (p)ppGpp. The current model of SR regulation posits that in this state, Rel exists as a dimer, and the TGS domain interacts with the NTD to maintain Rel in the Hd_on_/Syn_off_ state (4 – 6). However, in the setting of amino acid starvation, the C-terminal domains of Rel specifically recognize and bind to stalled ribosome complexes as a monomer. This causes Rel to undergo a conformational change that releases TGS repression on the NTD and transitions Rel to the Hd_off_/Syn_on_ state, thereby triggering net synthesis of (p)ppGpp.

*Staphylococcus aureus* is a clinically important pathogen in which Rel plays a key role in pathogenicity and virulence (1, 7 – 9). For *S. aureus*, the synthetase activity of Rel has been shown to be triggered by branched-chain amino acid [BCAA (isoleucine, leucine, valine)] starvation (7), although presumably amino acid limitation in general can elicit the SR. The synthesized (p)ppGpp is able to bind directly to various proteins to modulate their activities, such as those involved in ribosome assembly (10 – 13). Importantly, in *S. aureus*, the SR is tightly linked to the CodY regulon, which also responds to low BCAA levels. CodY is a transcriptional repressor that regulates hundreds of genes encoding major metabolism and virulence pathways, including genes critical for BCAA import and biosynthesis (14, 15). CodY binds to BCAAs and GTP, both of which activate its repressor activity. When BCAA levels are low, the resultant (p)ppGpp synthesis activity of Rel also causes a drop in GTP concentration. This inactivates CodY repressor activity and leads to the expression of CodY genes. In addition, low levels of GTP downregulate GTP-sensitive rRNA promoters, which slows protein translation (16, 17). Notably, *S. aureus* strains with *rel* mutations that inactivate its synthetase activity (*rel*_syn_) exhibit reduced virulence in a murine kidney infection model (7) and are compromised in their ability to escape from the phagolysosome of macrophages (8). Similarly, deletion of *codY* increases *S. aureus* virulence in murine models of necrotizing pneumonia and dermonecrosis (18). Thus, together, Rel and CodY constitute a cellular barometer for overall energy status and link metabolism with virulence (19).

We recently identified five novel *rel* mutations by analyzing within-host bacterial evolution in patients with persistent bacteremia caused by methicillin-resistant *S. aureus* (MRSA) (Table S1) (20). A prior study of two different clinically derived *rel* mutations showed that they caused partial activation of the SR and led to an antibiotic tolerance phenotype (21, 22). In the present study, we set out to characterize our additional five *rel* mutations and link them to mechanisms of persistent clinical infections. We show that our *rel* mutations impart both a survival and competitive growth advantage under conditions of nutrient stress due to increased SR activity, specifically in stationary phase growth. In contrast to the previously characterized mutations, the mutations we examined did not cause multidrug antibiotic tolerance. These findings demonstrate that clinical *rel* mutations can be selected for by a mechanism that is independent of antibiotic stress and highlight the multifaceted role of SR signaling in bacterial survival and pathogenesis during human infection.

## RESULTS

### Clinical *rel* mutations map to different protein domains

Two previous clinical *rel* mutations have been reported. One was identified in a case of persistent MRSA bacteremia and caused an F128Y substitution (21). The second was identified in a case of persistent *Enterococcus faecium* bacteremia and caused an L152F substitution (22). Both mutations map to the Hd subdomain and cause a (p)ppGpp hydrolysis defect, resulting in partial activation of the SR and increased antibiotic tolerance (21 – 24). In contrast, the five clinical *rel* mutations we identified are distributed throughout different Rel domains (Fig. 1A) (20). All five mutations are protein-altering. Four are nonsynonymous (D134Y, A301T, E384K, V670G), and the fifth is a 4-bp deletion encompassing codon N697 that results in a frameshift causing a premature stop codon at position 701 (herein denoted as N697* for convenience). N697* has a 29-amino acid truncation of the C-terminal ACT domain.

Mapping our mutations onto published crystal structures of *Bacillus subtilis* Rel suggested possible effects on Rel function (Fig. 1B) (5). First, D134Y, along with the previously identified F128Y and L152F substitutions, localize either to or near the central 3-helix bundle within the Hd subdomain, a region important for catalyzing (p)ppGpp hydrolysis (3). This suggested that D134Y may also result in impaired hydrolysis and an activated SR. Second, A301T and E384K localize to the surface of the Syn subdomain, adjacent to regions that interact with the TGS domain. This suggested that they may disrupt TGS repression of NTD synthesis activity, also leading to an activated SR (5). Finally, the remaining two mutations, V670G and N697*, lie within the C-terminal ACT domain. The ACT domain is important in recognizing and binding to stalled ribosome complexes during activation of the SR, and its deletion has been shown to result in over-production of (p)ppGpp due to dysregulation of enzymatic activity by the ribosome (25). This suggested that our ACT mutations may also lead to increased (p)ppGpp production. Overall, this analysis indicated that all five of our clinical *rel* mutations may cause SR activation, but through multiple different mechanisms.

### Clinical *rel* mutations alter bacterial growth kinetics in a response-dependent manner

To examine the specific phenotypic effects of the five mutations, we attempted to introduce each mutation into the MRSA USA300 JE2 strain by markerless allelic exchange. We were successful in creating mutants encoding the D134Y, E384K, and V670G amino acid substitutions but were unable to introduce the A301T and N697* variants despite numerous attempts. Possibly, these mutations impart a significant fitness cost that favors retention of the wild-type (WT) allele under the experimental conditions. Instead, we elected to create a complete ACT domain truncation mutant (ΔACT) as a surrogate to investigate its role in SR signaling. This yielded a panel of five *rel* strains in the JE2 background (herein referred to as WT, D134Y, E384K, V670G, and ΔACT).

To assess SR signaling for this panel of strains, we first determined their minimal inhibitory concentration (MIC) values for the antibiotic mupirocin. Mupirocin is a specific inducer of Rel (p)ppGpp synthetase activity in *S. aureus*. It selectively inhibits bacterial isoleucyl-tRNA synthetase, resulting in the accumulation of uncharged tRNA^Ile^, thereby mimicking isoleucine starvation. Notably, *rel*_syn_ mutants, which are defective in SR signaling, are known to exhibit a 4-fold reduction in mupirocin MIC (7). We found that the MIC of the WT strain was 0.25 μg/mL and the mutant *rel* strains were similar, ranging from 0.25 to 0.50 μg/mL (Table 1; Table S2). This demonstrated that Rel signaling remained intact in the strains harboring the clinical mutations.

**TABLE 1:** MIC data from the strain panel^*a*^

Strain	Vancomycin	Daptomycin	Ceftaroline	Mupirocin
WT	2 (2, 2, 2, 2, 2, 2, 2)	2 (1, 1, 2, 2, 2, 2, 2)	0.25 (0.25, 0.25, 0.25, 0.25, 0.25, 0.25, 0.25)	0.25 (0.25, 0.25, 0.25, 0.25, 0.25)
D134Y	2 (2, 2, 2, 2, 2)	2 (1, 1, 2, 2, 2)	0.25 (0.25, 0.25, 0.25, 0.25, 0.25)	0.25 (0.25, 0.25, 0.25, 0.25)
E384K	2 (2, 2, 2, 2, 2, 2)	2 (2, 2, 2, 2, 2, 2)	0.5 (0.5, 0.5, 0.5, 0.5, 0.25, 0.25)	0.5 (0.5, 0.5, 0.5, 0.5, 0.25, 0.25)
V670G	2 (2, 2, 2, 2, 2, 2)	2 (1, 1, 2, 2, 2, 2)	0.25 (0.25, 0.25, 0.25, 0.25, 0.25, 0.25)	0.25 (0.25, 0.25, 0.25, 0.25)
ΔACT	2 (2, 2, 2, 2)	2 (1, 1, 2, 2)	0.25 (0.25, 0.25, 0.25, 0.25)	0.25 (0.25, 0.25)

^*a*^ Values (μg/mL) from replicate (*N* = 2–7) MIC determinations are given inside parentheses. The mode value is given to the left outside parentheses, with the largest mode value reported if multimodal. Statistical analysis revealed no significant strain-to-strain differences (see Table S2).

**FIG 2:**
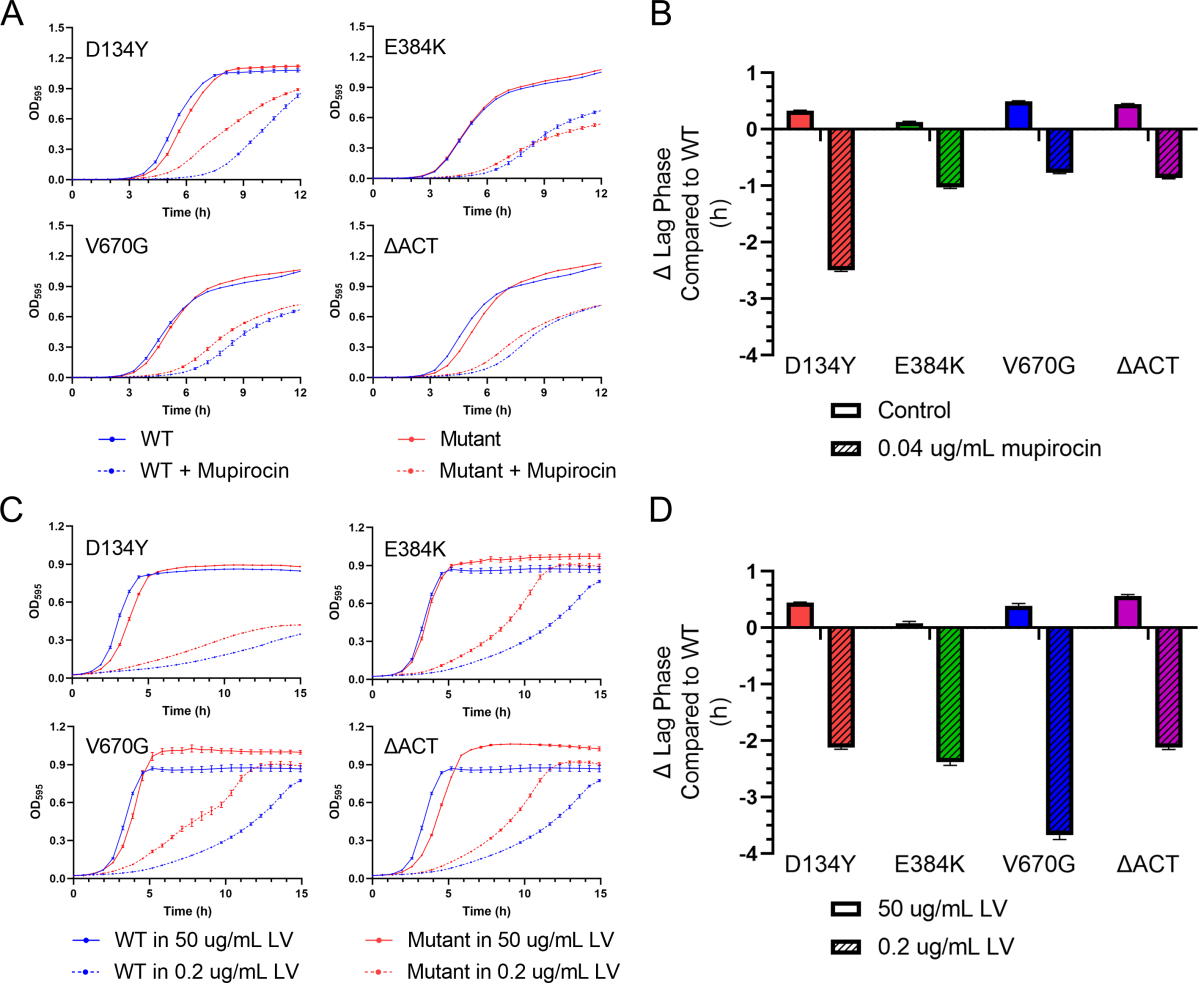
Rel mutants have a shorter lag phase when grown under nutrient stress. Growth curves (**A**) and lag phase comparisons (**B**) of WT and mutant strains grown in rich media challenged with a sub-MIC concentration of mupirocin (0.04 μg/mL). Growth curves (**C**) and lag phase comparisons (**D**) of WT and mutant strains grown in CDM containing either 50 or 0.2 μg/mL of LV. Growth curve data points and errors represent the mean and SEM of at least four independent biologic replicates, respectively. Data points and error of lag phase comparisons represent the best-fit value and 95% confidence interval from non-linear regression of at least three independent biologic replicates, respectively. No 95% confidence intervals cross zero, indicating that the calculated differences from WT are statistically significant. Growth variations in CDM are attributed to experiments being performed on different days using different medium batches.

To probe further, we next monitored growth kinetics in the absence or presence of a sub-inhibitory concentration of mupirocin (0.04 μg/mL), equivalent to 0.16× MIC. In the absence of mupirocin, all four mutant *rel* strains displayed a slightly longer lag phase than the WT strain. The V670G strain exhibited the largest effect, with an increase of 0.5 h. In contrast, in the presence of mupirocin, the mutant *rel* strains displayed a markedly shorter lag phase than WT (Fig. 2A and B). The D134Y strain had the most pronounced mupirocin effect, with a lag phase 2.5 h shorter than WT. To assess the specificity of this effect, we measured growth kinetics in the presence of several clinically relevant anti-MRSA antibiotics, also at a concentration equivalent to 0.16× MIC. We found that vancomycin, ceftaroline, and daptomycin caused only minor differences (<0.4 h) in lag phase between the WT and mutant *rel* strains (Fig. S1 and S2). These results show that the *rel* mutations reduce the duration of the lag phase in a mupirocin-specific manner.

### Clinical *rel* mutations impart a fitness advantage under nutrient stress

The SR in *S. aureus* is naturally induced by BCAA starvation and can be elicited with media containing low concentrations of leucine and valine (LV) (7, 8). Therefore, we next challenged our panel of *rel* mutants with BCAA stress. Growth was monitored in a chemically defined medium (CDM) that either contained an excess amount of LV (50 μg/mL) or a growth-limiting amount of LV (0.2 μg/mL). The results were similar to the mupirocin experiment described above. As compared to WT, all four mutant *rel* strains displayed an increased time in lag phase with excess LV and a decreased time in lag phase under limiting LV (Fig. 2C and D). In this experiment, however, the V670G strain showed the most pronounced effect of nutrient limitation, with a lag phase over 3.5 h shorter than WT.

**FIG 3:**
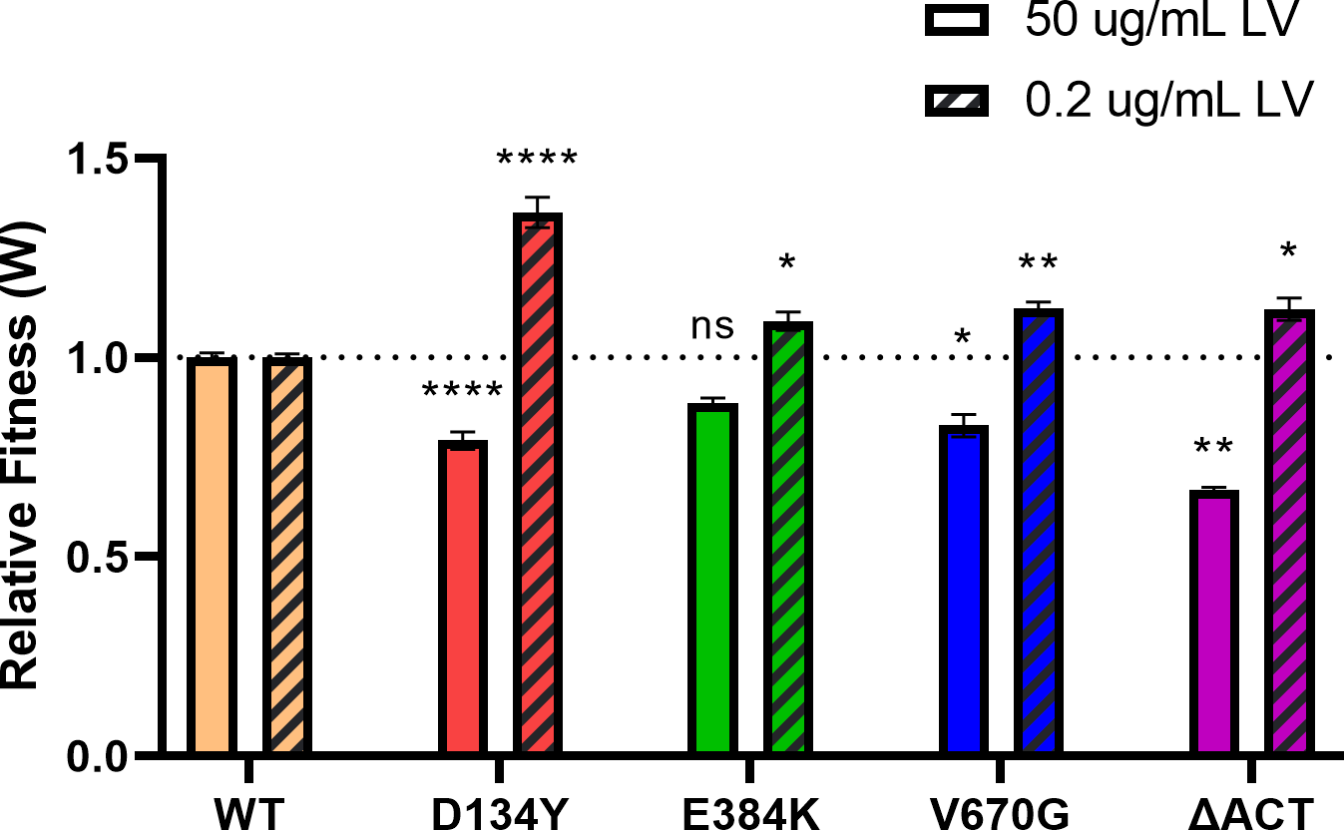
Rel mutants have a growth fitness advantage under nutrient stress. Normalized relative fitness (*W*) of mutant strains compared to WT, each competing with WT-GFP in CDM containing 50 or 0.2 μg/mL LV. A relative fitness value of 1.0, marked by the dotted line, denotes equal fitness with WT. Data points and errors represent the mean and SEM of three independent biologic replicates, respectively. Mean values were compared with the WT/WT-GFP control using an unpaired *t*-test (ns, not significant; *, *P* ≤ 0.05; **, *P* ≤ 0.01; ****, *P* ≤ 0.0001).

We next assessed whether the altered growth kinetics we observed would translate into fitness differences. To do this, we measured the relative fitness (*W*) of each mutant *rel* strain by co-culturing it with the WT strain in a competitive growth assay. We found that under conditions of excess LV, all of the mutations imparted a fitness cost, although the effect of E384K was not statistically significant (Fig. 3; Table S3). In contrast, under limiting amounts of LV, the pattern was reversed, with all of the mutants now displaying a significant fitness advantage. The D134Y strain demonstrated the greatest effect under these conditions (*W* = 1.36). These results show that the clinical *rel* mutations alter SR signaling in a manner that causes a fitness trade-off, with the advantage occurring only under nutrient-limiting conditions. Interestingly, the D134Y, E384K, and ΔACT mutants also displayed a slightly faster maximum growth rate than WT in the low LV condition (Fig. S3), so this may also contribute to the fitness advantage observed for these mutants, albeit to a lesser extent than the effect on lag phase.

### Clinical *rel* mutations that do not impart multidrug tolerance

**FIG 4:**
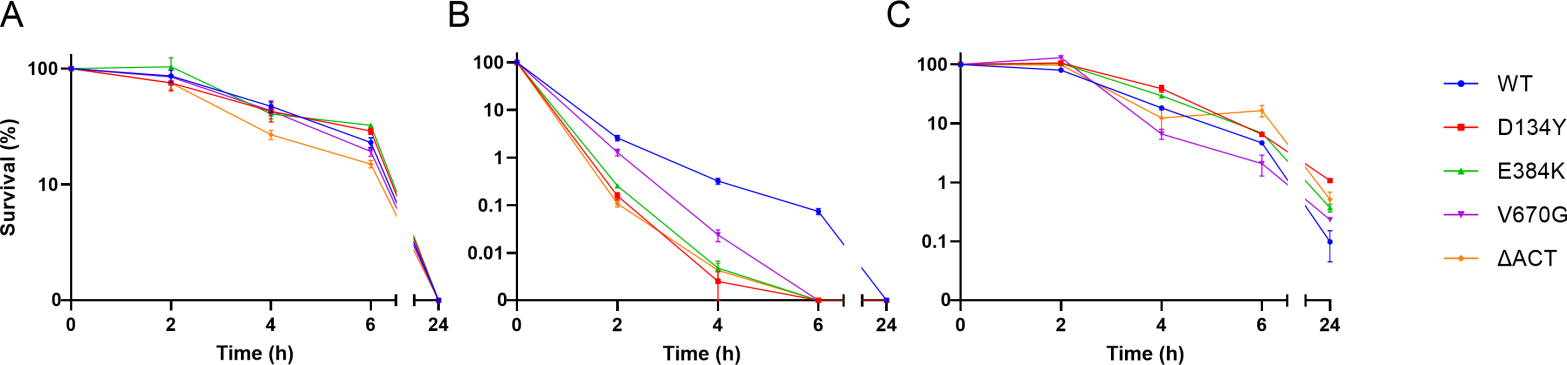
Clinical Rel mutants do not impart multidrug tolerance. Planktonic lag phase cultures of WT and Rel mutant strains were exposed to 4× the MIC of (**A**) vancomycin, (**B**) daptomycin, and (**C**) ceftaroline. The percentage of survival of the initial starting population was determined at set intervals. Data points and errors represent the mean and SEM of three independent biologic replicates, respectively.

Activation of the SR is classically linked to antibiotic tolerance by virtue of its effects on metabolism (10, 21, 22, 26, 27). Reduced rates of replication, transcription, and translation limit the efficacy of numerous classes of antibiotics, resulting in multidrug tolerance. To assess whether our clinical *rel* mutations convey antibiotic tolerance, we first determined the MIC of each mutant strain to several clinically relevant anti-MRSA antibiotics (Table 1; Table S2). As expected, none of the mutations resulted in a change in MIC. We next performed time-kill assays with vancomycin, daptomycin, and ceftaroline using antibiotic concentrations of 4× MIC as previously described (Fig. 4) (23) and used these data to calculate values for the minimum duration of killing (MDK) (Table 2). In contrast to prior reports, we found that our *rel* mutations did not impart multidrug tolerance. For vancomycin, there were no significant differences in MDK values between the four mutant strains and WT, and for ceftaroline, the kill kinetics of the mutants were heterogeneous compared to WT, with only D134Y and E384K displaying MDK values that were slightly greater than WT by ~0.5 h. Interestingly, for daptomycin, all four of the *rel* mutations actually caused decreased tolerance, with MDK values that ranged from 0.8 to 1.4 h less than WT. We also performed time-kill assays on overnight cultures as previously described (20), and once again, our mutants did not demonstrate multidrug tolerance (Fig. S4). Thus, in contrast to the two previously identified clinical *rel* mutations, these mutations do not impart antibiotic tolerance.

**TABLE 2:** Minimal duration of killing*^a^*

Strain	MDK (−95% CL, +95% CL) (h)		
	VAN MDK_50_	DAP MDK_99_	CPT MDK_75_
WT	3.7 (3.1, 4.2)	3.0 (2.7, 3.4)	3.6 (3.4, 3.8)
D134Y	3.5 (2.4, 4.3)	1.6 (1.3, 1.8)*	4.2 (3.9, 4.6)*
E384K	4.0 (3.1, 5.1)	1.7 (1.4, 2.0)*	4.1 (4.0, 4.3)*
V670G	3.5 (3.2, 3.8)	2.2 (2.0, 2.3)*	3.3 (2.6, 3.8)
ΔACT	2.8 (2.2, 3.3)	1.6 (1.2, 1.9)*	~4

^*a*^ Values were determined from the data shown in Fig. 4. The 50% (MDK50), 99% (MDK99), and 75% (MDK75) survival thresholds for vancomycin (VAN), daptomycin (DAP), and ceftaroline (CPT), respectively, were chosen based on the different kill kinetics observed within 6 h to ensure that calculations were based on the linear range of the assay for all strains. Numbers in parentheses represent 95% confidence limits (CLs) derived from interpolated best-fit values from linear regression using all replicate values (*n* = 3) from three different time points. Asterisks (*) denote non-overlapping 95% CLs with the WT strain. CLs could not be estimated for the ACT strain with CPT due to the nonlinear behavior of killing.

### Clinical *rel* mutations partially activate the SR during the stationary phase of growth

To better understand the lag phase-specific growth effect and the lack of an antibiotic tolerance phenotype for our *rel* mutations, we next set out to quantify the ability of the mutant *rel* strains to generate (p)ppGpp. Prior studies of SR-activating *rel* mutations have quantified (p)ppGpp levels during the exponential phase and found increased levels under these conditions even under nutrient-rich conditions (7, 21 – 23). Therefore, using mass spectrometry, we first quantified intracellular levels of GTP, ppGpp, and pppGpp during exponential growth in nutrient-rich media. As a positive control, parallel cultures were also induced with an inhibitory amount of mupirocin (0.3 μg/mL). We found that the fraction of (p)ppGpp in the guanine nucleotide pool of the mutant strains was either equivalent (D134Y) or less (E384K, V670G, ΔACT) than WT (Fig. S5; Tables S4 and S5). Furthermore, with the mupirocin challenge, only E384K and V670G had (p)ppGpp levels greater than WT, whereas D134Y and ΔACT did not. Thus, in contrast to prior studies, a consistent signal of heightened SR activation was not observed for our mutant *rel* strains. This suggested that either intracellular (p)ppGpp is not responsible for the lag phase phenotype we observed or, more likely, (p)ppGpp levels are context-dependent.

**FIG 5:**
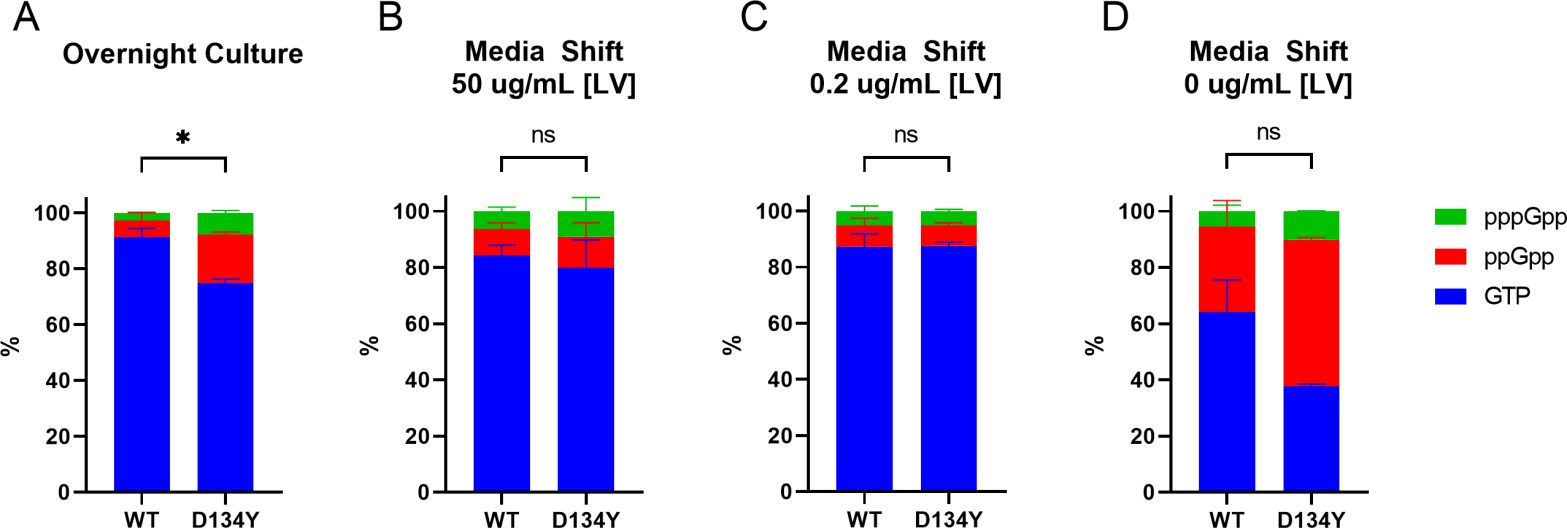
*Rel* mutations activate the stringent response during the stationary phase. UPLC-MS/MS quantification of intracellular GTP, ppGpp, and pppGpp of (**A**) overnight cultures grown in TSB, and overnight cultures grown in TSB subsequently media-shifted into (**B**) 50, (**C**) 0.2, and (**D**) 0 μg/mL LV CDM. Quantification is displayed as % composition of the quantified guanosine nucleotide pool (GTP + ppGpp + pppGp). Data points and errors represent the mean and SEM of three independent biological replicates, respectively. Mean values of % GTP were compared using an unpaired *t*-test (ns, not significant; *, *P* ≤ 0.05).

Therefore, instead of measuring (p)ppGpp during exponential phase growth in nutrient-rich media, we measured (p)ppGpp levels under the same conditions as our growth assays. Notably, the growth assays were initiated using cells in overnight culture. Thus, for this analysis, we measured (p)ppGpp levels of the WT and D134Y strains from overnight cultures and also in cells that were media-shifted from the overnight cultures into either CDM containing excess LV (50 μg/mL) or limiting LV (0.2 μg/mL). As a control for SR activation, we also included cells shifted into CDM that completely lacked LV (0 μg/mL). We chose the D134Y strain for this additional analysis because it displayed the greatest fitness advantage in the competitive growth assay, yet it was the one mutant that failed to exhibit any increase in (p)ppGpp levels in measurements using nutrient-rich media. Interestingly, in overnight culture, we found that the mutant had a significantly higher fraction of (p)ppGpp than WT (Fig. 5A). However, when the medium was then shifted into either excess LV (Fig. 5B) or limiting LV (Fig. 5C), this difference was no longer present. The shift into 0 μg/mL LV media maintained higher (p)ppGpp levels, but the difference relative to WT was not statistically significant (Fig. 5D). Taken together, these results demonstrate that (p)ppGpp levels in the *rel* mutant strains are only measurably greater than those in the WT strain under certain stress contexts, such as mupirocin challenge (E384K, V670G) or overnight culture (D134Y), but not when nutrients are available.

### Clinical *rel* mutations convey a survival advantage during the prolonged stationary phase

**FIG 6:**
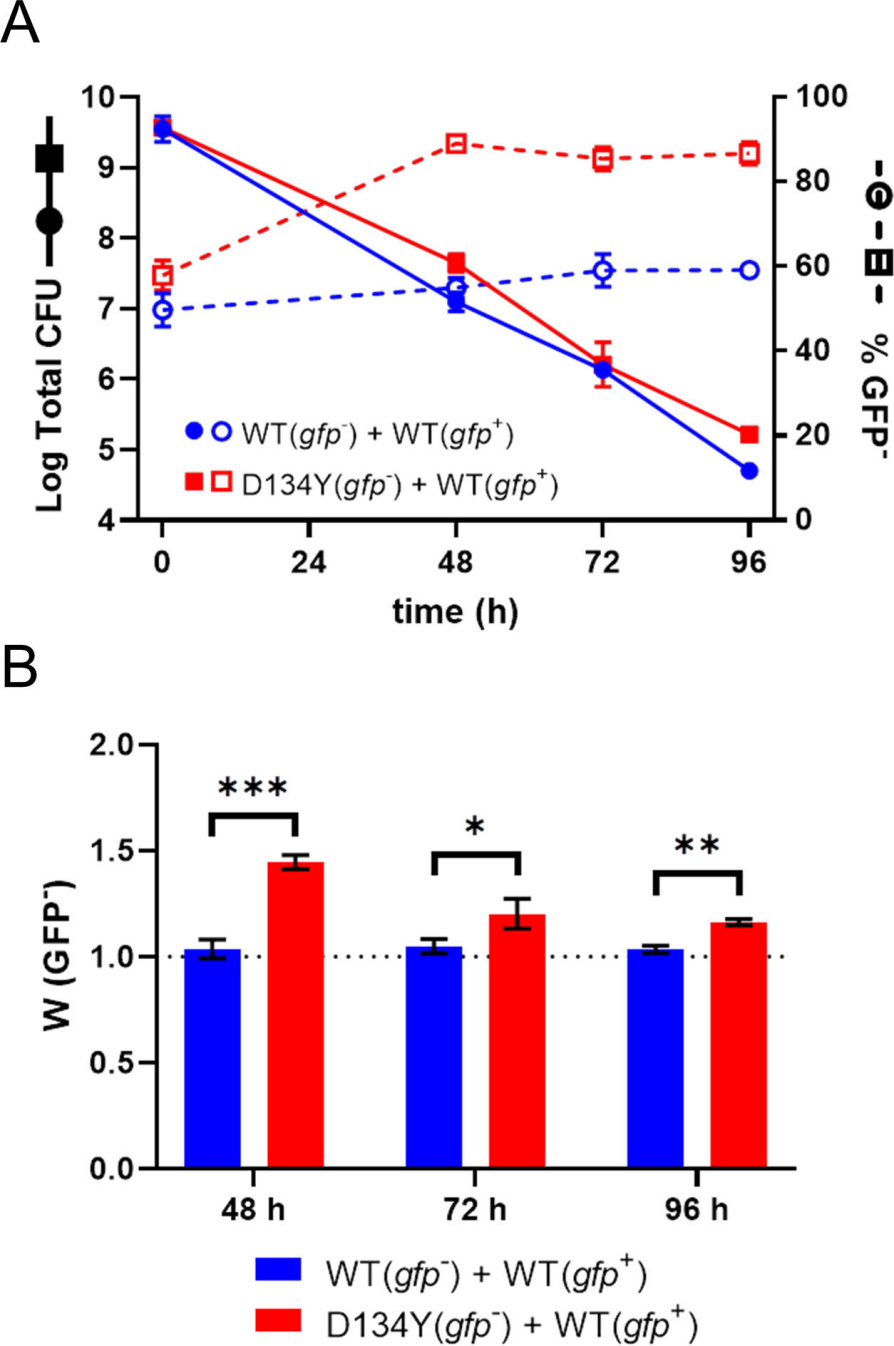
Rel mutants have a survival advantage under nutrient stress. (**A**) Survival of co-cultures of WT-GFP (*gfp^+^*) and either WT or D134Y (*gfp^−^*) during continuous stationary phase incubation. Data at each time point are represented by total population CFU and % *gfp^−^* cells in the surviving population. (**B**) Survival fitness of WT and D134Y (*gfp^−^*) relative to WT-GFP (*gfp^+^*) in co-culture at designated time points during continuous stationary phase incubation. A survival fitness value of 1.0, marked by the dotted line, denotes neither a survival advantage nor a survival disadvantage. Data points and errors represent the mean and SEM of three independent biological replicates, respectively. Comparisons were performed using an unpaired *t*-test (*, *P* ≤ 0.05; **, *P* ≤ 0.01; ***, *P* ≤ 0.001).

Bacteria enter the stationary phase when nutrition becomes limiting, activating numerous stress response systems, including the SR, to reprogram cellular physiology in order to adapt to increasingly harsh conditions (28). Therefore, based on our findings of (p)ppGpp differences within overnight cultures, we hypothesized that our mutants would have a survival advantage in a more prolonged stationary phase culture. To test this, we measured the relative fitness of the D134Y strain in co-culture with the WT strain using a competitive survival assay in spent CDM over 96 h. As expected, the number of viable cells decreased over time (Fig. 6A). Notably, however, the mutant had a consistently higher rate of survival at each time point (Fig. 6A), which translated into a significantly elevated value for relative fitness throughout the experiment (Fig. 6B). Bacterial inoculation into filter-sterilized media from the 48-h time point showed that the medium does not support detectable growth at this time, suggesting that the increased survival is due to a decreased rate of death rather than increased cellular replication (Fig. S6). Together, these findings link the increased SR activation during the stationary phase to a survival advantage in a nutritionally depleted environment.

## DISCUSSION

Nutrient availability plays an important role in multiple facets of the bacterial host-pathogen interaction. To gain a foothold within the host, pathogens must first overcome restrictive nutritional access within the microenvironment imposed by the host’s own microbiome (29 – 31). After establishing a colony, the pathogen must then successfully multiply in the face of numerous host mechanisms of nutrient sequestration (32 – 34). Furthermore, pathogens engulfed by professional phagocytes are challenged with various nutriprive mechanisms that target phagocytosed bacteria (35, 36). These various strategies, the latter two of which are collectively termed “nutritional immunity,” serve as important nutrient-centered host defenses against bacterial pathogens.

*S. aureus* is both a human commensal and an opportunistic pathogen able to cause invasive infections with significant morbidity and mortality. Colonization is linked to the development of these invasive infections (37 – 39), and a recent WGS study identified within-host evolutionary pathways connecting colonizing and invasive phenotypes (40). The ability for *S. aureus* to thrive in such divergent niches within the human host highlights its ability to rapidly modulate its own transcriptome to adapt to dynamic environmental conditions—including fluctuating nutrient status—to survive, proliferate, and evade host immune responses. Central to this flexibility are two global regulators: the SR and the CodY regulon (41, 42). In the face of BCAA limitation, both systems work in tandem to release the expression of hundreds of metabolism and virulence genes to allow *S. aureus* to overcome hostile environments.

SR activation and CodY regulon de-repression naturally occur during the stationary phase to facilitate adaptation (28). In this study, we have characterized *rel* mutations that further increase stationary phase (p)ppGpp alarmone production, resulting in both enhanced survival and subsequent expansion under nutrient-limiting conditions. The growth advantage is primarily due to a shorter lag phase. These findings suggest that our *rel* mutations augment the existing adaptive programming to not only improve overall survival but also facilitate pathogen expansion within the host. Our findings are also consistent with a recent study highlighting the importance of the SR in priming bacteria for success when shifted to a nutritionally poor medium (43). The underlying mechanism for the observed fitness advantages of our mutants in low-nutrient media can likely be attributed to upregulation of amino acid transport and metabolism pathways, as previously demonstrated (8, 43, 44). Conversely, this may also explain the observed fitness disadvantage in nutrient-rich media, as a heightened SR in the absence of any true nutrient deficiencies would only serve to mistakenly redirect cellular programming and impair overall cellular growth.

Importantly, our findings suggest that selective pressures exist within patients during persistent infection that favor an augmented SR. One potential niche that may have led to this within-host evolution is in macrophage phagolysosomes. Phagocytosed *S. aureus* is a major source of recurrent infection (45), and intracellular populations have been shown to possess antibiotic tolerance (46 – 48), heightened stress responses, and increased expression of *rel* (49). The nutriprive pressures inside the phagolysosome may select for adaptive *rel* mutations that grant a survival advantage in this harsh environment. Another microenvironment that may have given rise to these *rel* mutations are the abscesses that often form in tissues during systemic *S. aureus* infection (50). *S. aureus* abscess collections are low in glucose but rich in L-lactate and peptides (51). However, nitric oxide from host phagocytes (52, 53) disrupts *S. aureus* metabolic enzymes and precludes the sole use of L-lactate for cellular metabolism and growth (54, 55). Thus, it has been proposed that *S. aureus* utilizes a metabolic partitioning strategy whereby L-lactate is the predominant source of ATP, which, in turn, facilitates amino acid import to provide carbon sources for cellular growth and biomass (54). Given that the CodY regulon controls numerous genes involved in amino acid transport (8), activation of the SR and subsequent de-repression of CodY would certainly enhance *S. aureus* growth within these abscess communities.

Strikingly, our *rel* mutations, unlike those previously identified, do not impart multidrug tolerance. The association between an activated SR and a tolerance phenotype is well established in both gram-positive and gram-negative bacteria (56). Furthermore, *rel* mutations engineered into either methicillin-sensitive *S. aureus* strain Newman (23) or MRSA strain J01 (57) have demonstrated increased antibiotic tolerance. And yet, despite performing time-kill assays in both lag and stationary phase cultures using established experimental protocols (20, 23), we failed to uncover a tolerance phenotype. One possibility for this discrepancy is different strain backgrounds, as we used MRSA strain JE2. However, we note that another possibility is varying degrees or conditions of heightened SR activation. The SR is not a binary switch but rather a titratable system in which different cellular processes are mediated by a gradient of intracellular (p)ppGpp concentrations that is tightly regulated (2). The *rel* mutations studied here may result in a rate of net (p)ppGpp synthesis that grants a nutritional growth advantage but is insufficient to convey antibiotic tolerance. For example, we identified evidence of increased intracellular (p)ppGpp during the stationary phase (Fig. 5A), but this did not translate to multidrug tolerance (Fig. S4). This may also explain the heterogeneity we observed in our panel’s response to antibiotic challenge, in particular the increased sensitivity to daptomycin-mediated killing. Since (p)ppGpp levels mediate the stability of cellular membranes (58), the increased SR signaling during the stationary phase could have altered membrane integrity, resulting in an unexpected increase in daptomycin susceptibility during the lag phase (Fig. 4B). We also note that it is possible that they cause dysregulation more specifically under stressful conditions. This could serve to reduce the degree of fitness trade-off that occurs under nutrient-rich conditions. Consistent with this, we found that our mutant *rel* strains only accumulated (p)ppGpp to levels greater than WT when challenged with stress conditions that actually halted growth and not during unstressed basal conditions, whereas prior studies detected (p)ppGpp elevations even during the exponential phase of growth, a time when net (p)ppGpp synthesis must be great enough to also outpace its dilution due to rapid cell division.

Finally, it is notable that despite having Rel mutations located in different regions of the protein, they all behaved similarly in our assays. This convergent phenotype belies the complex regulation of Rel and the distinct yet complementary roles of the various sub-domains in activation of the SR. Increased intracellular (p)ppGpp may be due to enhanced synthetase activity, decreased hydrolase activity, altered ribosome binding, or a combination of these factors. It is impossible to deduce the exact molecular mechanism of our mutations using cellular assays alone, but the mutations themselves can serve as tools to probe the system. For instance, WT and mutant Rel proteins can be used in enzyme kinetic assays to compare and contrast the activities of the hydrolase and synthetase domains. Linking protein structure and function, intracellular (p)ppGpp levels, and cellular phenotype may reveal additional insight into Rel and the SR.

In conclusion, we have characterized a more subtle class of clinical *rel* mutants that likely did not arise due to antibiotic selection but rather as an adaptation to combat host nutritional immunity. Our findings show that the evolution of SR-activating strains encompasses a broader range of adaptive SR phenotypes than previously appreciated, and that the underlying molecular mechanisms can be due to perturbing Rel’s regulatory domains in addition to its enzymatic domain. Our findings support Rel in facilitating adaptation of bacteria to different nutritional niches (59), and our novel *rel* mutations will serve as tools in understanding its complex regulation. Elucidating the specific mechanisms and pathways involved may provide valuable insight into the development of anti-metabolism therapeutics to serve as adjunctive therapies alongside traditional antibiotics.

## MATERIALS AND METHODS

### Bacterial strains

The *S. aureus* USA300 LAC strain JE2 and its (p)ppGpp^0^ (*Δrel ΔrelP ΔrelQ*) derivative were kindly provided by Dr. Christiane Wolz (60). Specific *rel* mutations were introduced into wild-type JE2 by markerless allelic exchange using the vector pIMAY-Z (61). For the D134Y, E384K, and V670G strains, DNA fragments containing at least 500 bp flanking the site of mutation in *rel* were amplified from the clinical isolate (20) using primers listed in Table S6 and cloned into pIMAY-Z using the EcoRI restriction site with HiFi DNA Assembly (New England Biolabs). For the ΔACT strain, a fragment was first amplified from WT genomic DNA using primers MC480/MC481 and cloned into pIMAY-Z using HiFi DNA Assembly as above, and then a nonsense mutation was introduced with primers EC134/EC135 using Q5 Site-Directed Mutagenesis (New England Biolabs). Turbo Competent *Escherichia coli* (New England Biolabs) were used for the cloning, and the desired sequences of the pIMAY-Z allelic exchange vectors were confirmed by Sanger sequencing (Genewiz). The *E. coli* DH10B derivative strain IM08B was used to prepare constructed pIMAY-Z plasmid DNA for direct transformation into *S. aureus* (62), and allelic exchange was carried out as previously described (61). The *gfp^+^* of JE2 was created exactly as previously described using the *gfp* integration vector pTH100 (63). All strains and desired mutations were validated by whole-genome sequencing. Illumina reads were aligned to the JE2 reference sequence (GenBank accession: NZ_CP020619.1) using *breseq* (64).

### Growth analysis

*S. aureus* strains were grown for 20 h at 37°C in 96-well plates (VWR 10861-562), and OD_595_ measurements were acquired every 3–5 minutes with programmed shaking between measurements using an Infinite F200 plate reader (Tecan). For growth in sub-MIC antibiotics, tryptic soy broth (TSB) was inoculated with an ~1,000-fold dilution of an overnight culture, with culture densities between strains normalized to the same OD_595_ value. Concentrations used for sub-MIC antibiotics are 0.32 μg/mL vancomycin, 0.32 μg/mL daptomycin, 0.04 μg/mL ceftaroline, and 0.04 μg/mL mupirocin. For growth in limiting and excess LV CDM (65), plates were inoculated with an ~100-fold dilution of an overnight culture with strains normalized to the same OD_595_ value. Inoculations for different growth conditions with the same strain were always from the same culture source to serve as an internal methodology control to account for colony-forming unit (CFU) variation. Lag phase duration from growth curves was determined by nonlinear regression using the grofit package (66), the Gompertz equation (sub-MIC growths), or the logistic equation (CDM growths) in Prism (GraphPad).

### Fitness assays

For competitive growth fitness assays, mutant *rel* strains (*gfp^−^*, dark) were competed against WT-GFP strains (*gfp* ^+^, bright) by overnight growth in co-culture. As a control, the *gfp^−^* parental WT strain was also competed against WT-GFP. First, individual overnight TSB cultures of the strains were pelleted, washed twice with normal saline (NS), and again resuspended in NS. The culture densities of each strain were measured at OD_595_ and then normalized and combined into a 14-mL culture tube containing 1 mL of the specified CDM at a final dilution of 1:100. Co-cultures were set up in triplicate and incubated for 20 h at 37°C with shaking at 250 RPM. The size of initial populations (*N*_*i*_) and final populations (*N*_*f*_) was determined by plating aliquots onto tryptic soy agar (TSA) and counting CFUs. CFUs of the bright and dark competitor strains were distinguished using a blue-light transilluminator. Raw relative fitness values (*W*) were first calculated for each replicate experiment using the following equation (67):

$W=ln\left(\frac{N_{f}^{dark}}{N_{i}^{dark}}\right)/ln\left(\frac{N_{f}^{bright}}{N_{i}^{bright}}\right)$ .

To normalize the relative fitness values between experiments performed on different days, the *W* values of each strain were then divided by the mean *W* value of the WT vs WT-GFP internal control performed on the same day.

For survival fitness assays, WT-GFP, WT, and D384Y strains were first cultured in 50 μg/mL LV CDM overnight for 16 h. Then, without any other manipulations, the WT-GFP strain was mixed 1:1 with either the WT or the D384Y strain in a 14-mL culture tube containing a final culture volume of 2 mL. The co-cultures were set up in triplicate and incubated at 37°C with shaking at 250 RPM. Initial and time-point population sizes were enumerated as above. A survival fitness value (*W*) was calculated using the inverse of the above equation.

### MIC determination

MICs were measured in cation-adjusted Mueller-Hinton broth supplemented with 50 μg/mL CaCl_2_ (MHB-Ca^2+^) via broth microdilution in 96-well plates with an inoculum of ∼5 × 10^5^ CFU per mL. Antibiotic test concentrations ranged from 0.0625 to 32 μg/mL by 2-fold dilutions. Plates were incubated at 37°C for 16 h without shaking, and results were interpreted in accordance with the European Committee on Antimicrobial Susceptibility Testing reading guidelines (68).

### Time-kill assays

Time-kill assays on planktonic lag phase bacterial cultures were performed as previously reported (23). Overnight bacterial cultures grown in TSB were diluted 1:1,000, in triplicate, into fresh MHB-Ca^2+^ containing 4× MIC of tested antibiotics and incubated shaking at 37°C and 250 RPM. Viable counts were determined on TSA following dilution in sterile NS, immediately after addition of antibiotic, and at 2-, 4-, 6-, and 24-h intervals. Time-kill assays on planktonic stationary-phase bacterial cultures were performed as previously reported (20). Overnight bacterial cultures were first grown in MHB-Ca^2+^ and split into triplicate cultures, to which 4× MICs of tested antibiotics were added, and incubated shaking at 37°C and 250 RPM. Viable counts were determined on TSA following dilution in sterile NS, immediately before addition of antibiotic, and at 72 h. All data shown on a single plot were performed at the same time using the same reagent stock.

### Mass spectrometry quantification of intracellular nucleotides

For nucleotide quantification of exponential phase growth, overnight (~16 h) cultures grown in TSB were freshly diluted into new TSB and grown until OD_595_ ~0.5 and harvested on a 0.22-μM vacuum filter; for the control, 0.3 μg/mL mupirocin was added, allowed to induce for 1 h shaking, and harvested on a 0.22-μM vacuum filter. For quantification of media-shifted conditions, overnight cultures grown in TSB were pelleted, washed with normal saline, resuspended in CDM, allowed to incubate shaking for 1 h, and harvested. Once harvested, the bacteria were washed with sterile, ice-cold water and resuspended in 400 μL of 100% methanol. The suspension was then transferred into a tube containing silica spheres (Lysing Matrix B, MP Biomedicals) and mechanically homogenized. Then, 400 μL each of 5 mM N,N-dimethylhexylamine (DHMA) pH 7.5 (Sigma) and 25:24:1 (vol/vol) phenol:chloroform:isoamyl alcohol (Sigma) was added and vortexed vigorously. The mixture was centrifuged to separate the layers, and the aqueous top layer was taken for quantification. Then, 50 µL of sample was used, and samples were further protein precipitated using 100 µL of methanol containing the internal standard (*d*14-GTP) before analysis via ultra-performance liquid chromatography tandem mass spectrometry (UPLC-MS/MS). The linear range of the standard curve was 25–5,000 ng/mL for all three analytes (ppGpp and pppGpp, Jena Bioscience; GTP, Cayman Chemicals). The UPLC-MS/MS system consists of a Thermo Scientific Vanquish UPLC and a Thermo Scientific TSQ Altis mass spectrometer equipped with a heated ESI (HESI) source. Chromatographic separation was performed using a Waters Acquity BEH phenyl column (2.1 × 150 mm, 1.7 µM) and a BEH phenyl VanGuard precolumn (2.1 × 5 mm). The analytes were separated under isocratic conditions (50:50, A:B) with a total runtime of 3.5 minutes using the mobile phases 5 mM DHMA at pH 7 in water (A) and 5 mM DHMA in acetonitrile at pH 7 (B). The analytes were detected using selected reaction monitoring (SRM) in negative ion mode (Fig. S7). The SRM transitions were *m/z* 521.9→424.0 for GTP, *m/z* 601.9→503.9 for ppGpp, and *m/z* 681.9→583.9 for pppGpp. The standard curve was created fresh for each run with duplicate QCs (low, medium, and high) to ensure that the results for each day were accurate (Fig. S8). The intraday accuracy and precision were assessed and were less than 11% for all QC levels for each of the analytes.

## Data Availability

All relevant data are contained within this article, presented either in the main body or in the supplemental materials. Further inquiries can be directed to the corresponding author.

## SUPPLEMENTAL MATERIAL

Supplemental Figures msphere.00249-23-s0001.pdf Fig. S1 to S8.

Supplemental Tables msphere.00249-23-s0002.docx Tables S1 to S6.

ASM does not own the copyrights to Supplemental Material that may be linked to, or accessed through, an article. The authors have granted ASM a non-exclusive, world-wide license to publish the Supplemental Material files. Please contact the corresponding author directly for reuse.
