# Supplementary material for: Clinical rel mutations in Staphylococcus aureus prime pathogen expansion under nutrient stress
Source: mSphere. 2023 Sep 26;8(5):e00249-23. doi: 10.1128/msphere.00249-23 (PMC10597345; doi:10.1128/msphere.00249-23)
Supplement: Supplemental Figures — Fig. S1 to S8. [file msphere.00249-23-s0001.pdf]

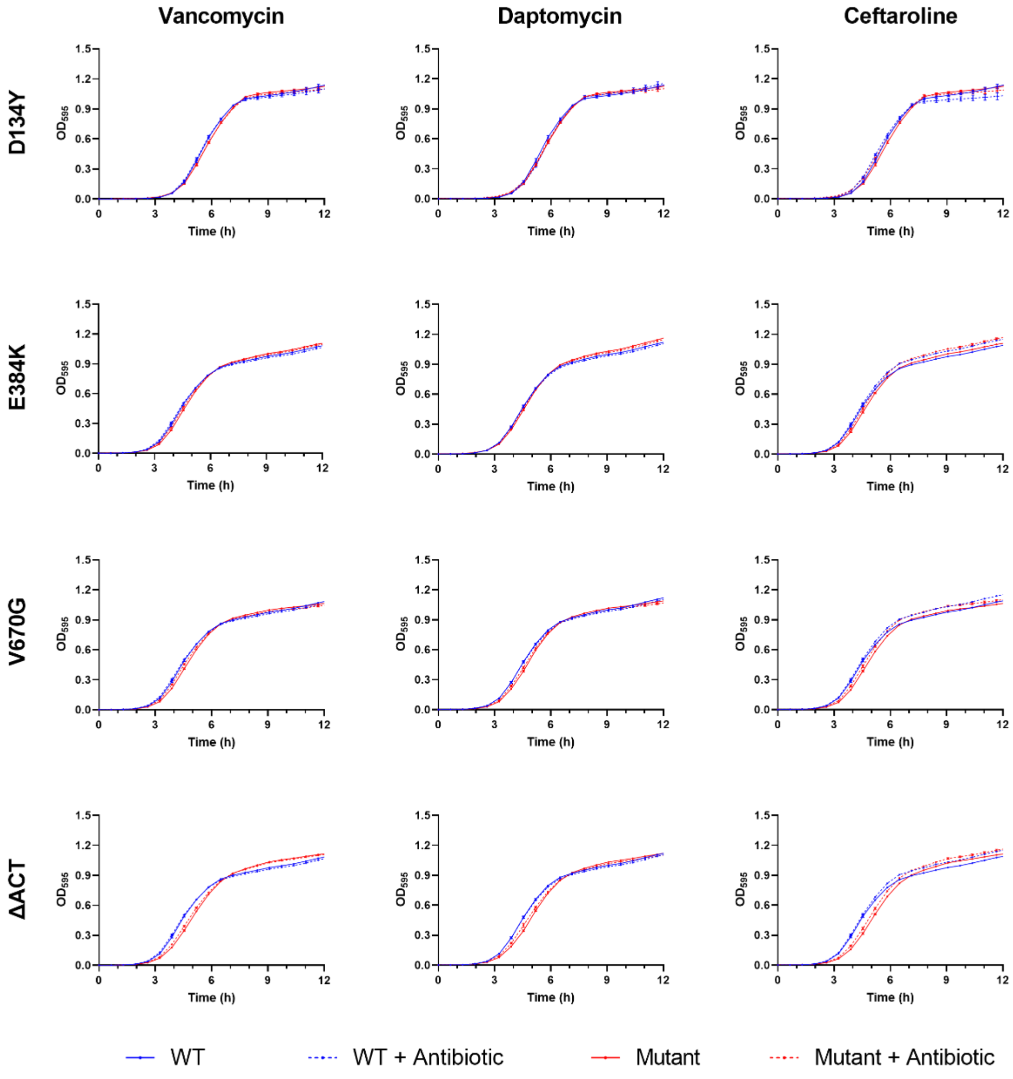

Fig S1 Growth analysis of mutant strains compared to WT when grown in rich media in the presence of sub-MIC concentrations of vancomycin (0.32 ug/mL), daptomycin (0.32 ug/mL), or ceftaroline (0.04 ug/mL). Data points and error represents the mean and SEM of at least four independent biologic replicates, respectively.

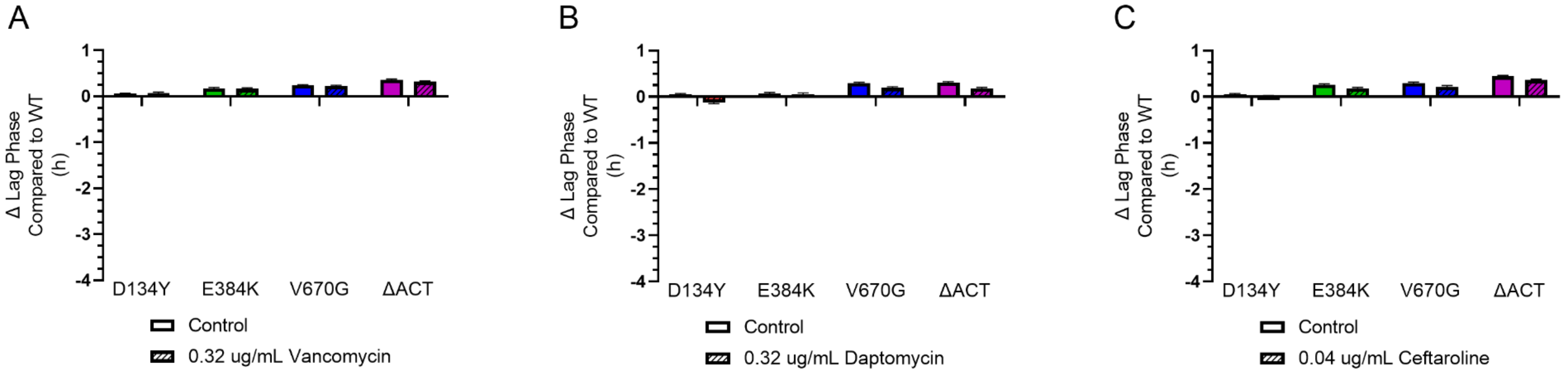

FIG S2 Lag phase of mutant strains are not affected by stringent response-independent antibiotics. Lag phase of Rel mutant strains compared to WT in rich media containing sub-MIC concentrations of (A) vancomycin, (B) daptomycin, and (C) ceftaroline. Data points and error represents the best-fit value and 95% confidence interval from non-linear regression of at least three independent biologic replicates, respectively.

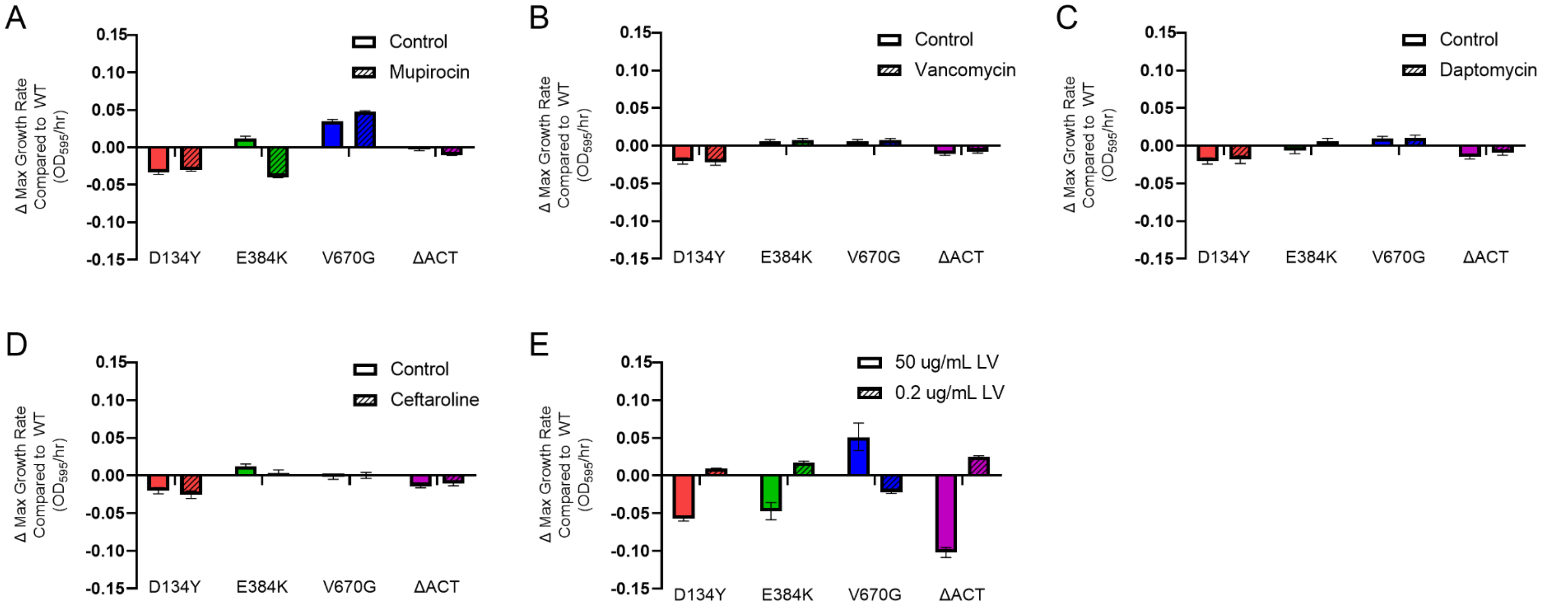

FIG S3 Fitness advantage of Rel mutants are not due to an increased growth rate. Max growth rates of Rel mutant strains compared to WT in rich media containing sub-MIC concentrations of (A) mupirocin, (B) vancomycin, (C) daptomycin, (D) ceftaroline, or, (E) in CDM containing either 50 ug/mL LV or 0.2 ug/mL LV. Data points and error represents the best-fit value and 95% confidence interval from non-linear regression of at least three independent biologic replicates, respectively.

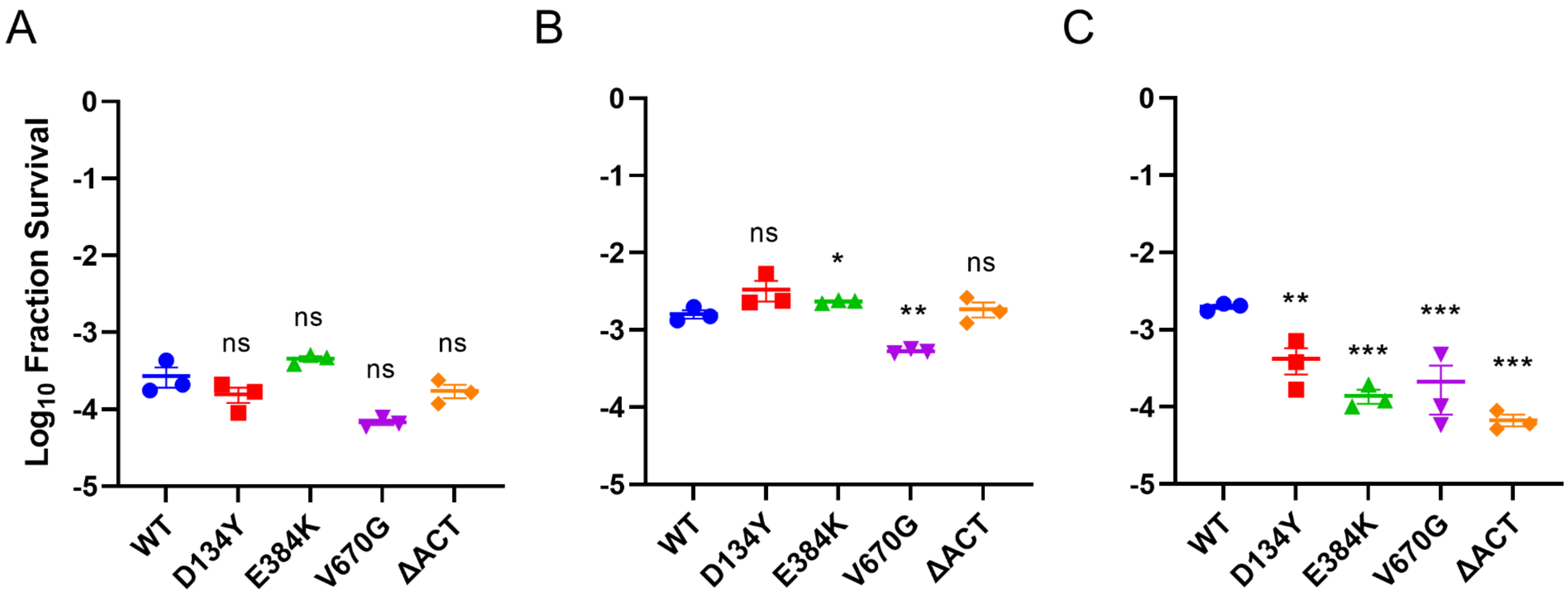

FIG S4 Clinical Rel mutants do not impart multidrug tolerance. Planktonic stationary phase cultures of WT and Rel mutants were exposed to 4x the MIC of (A) vancomycin, (B) daptomycin, and (C) ceftaroline. Percent survival of initial starting population was determined after 72 hours. Data points represent a scatter plot of three independent biologic replicates. The line and error bars represent mean values and SEM, respectively. Mean values of the mutants were compared to WT using unpaired t-test (ns, not significant; *, $P \leq 0.05$; **, $P \leq 0.01$; ***, $P \leq 0.001$).

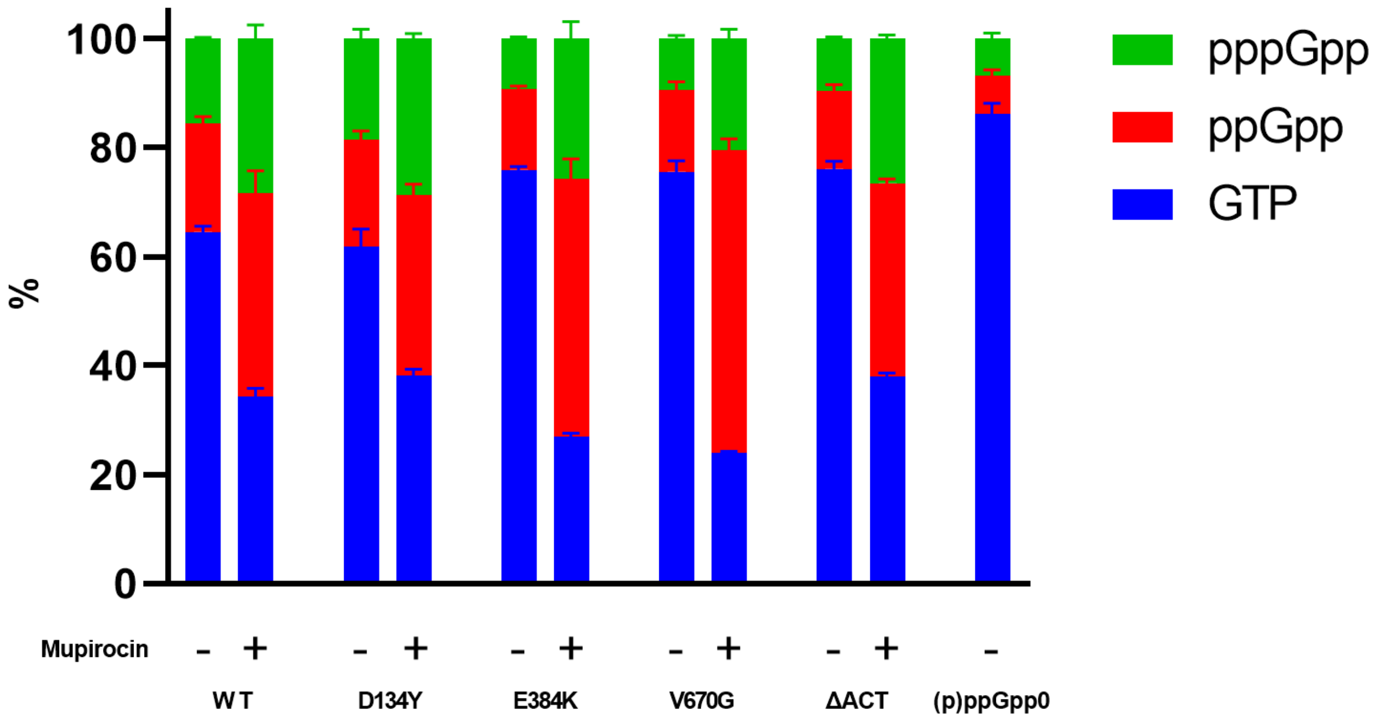

FIG S5 Quantification of intracellular GTP, ppGpp, and pppGpp at mid-exponential phase using UPLC-MS/MS and displayed as % composition of the quantified guanosine nucleotide pool (GTP + ppGpp + pppGpp). Mupirocin was added at 0.3 ug/mL as a stringent response inducing control. The (p)ppGpp^0 (Δrel ΔrelP ΔrelQ) strain has no ability to synthesize (p)ppGpp in an RSH-dependent manner and serves as a control for baseline detection. Note that the LC-MS signal peaks from the (p)ppGpp^0 strain for ppGpp and pppGpp detection exhibited signal-to-noise ratios of >10:1 and were interpreted as true peaks, which may represent low-level detection of (p)ppGpp isomers and/or RSH-independent sources of (p)ppGpp. Subtracting this background signal does not alter the statistical analysis or interpretation. Data points and error represents the mean and SEM of three independent biologic replicates, respectively.

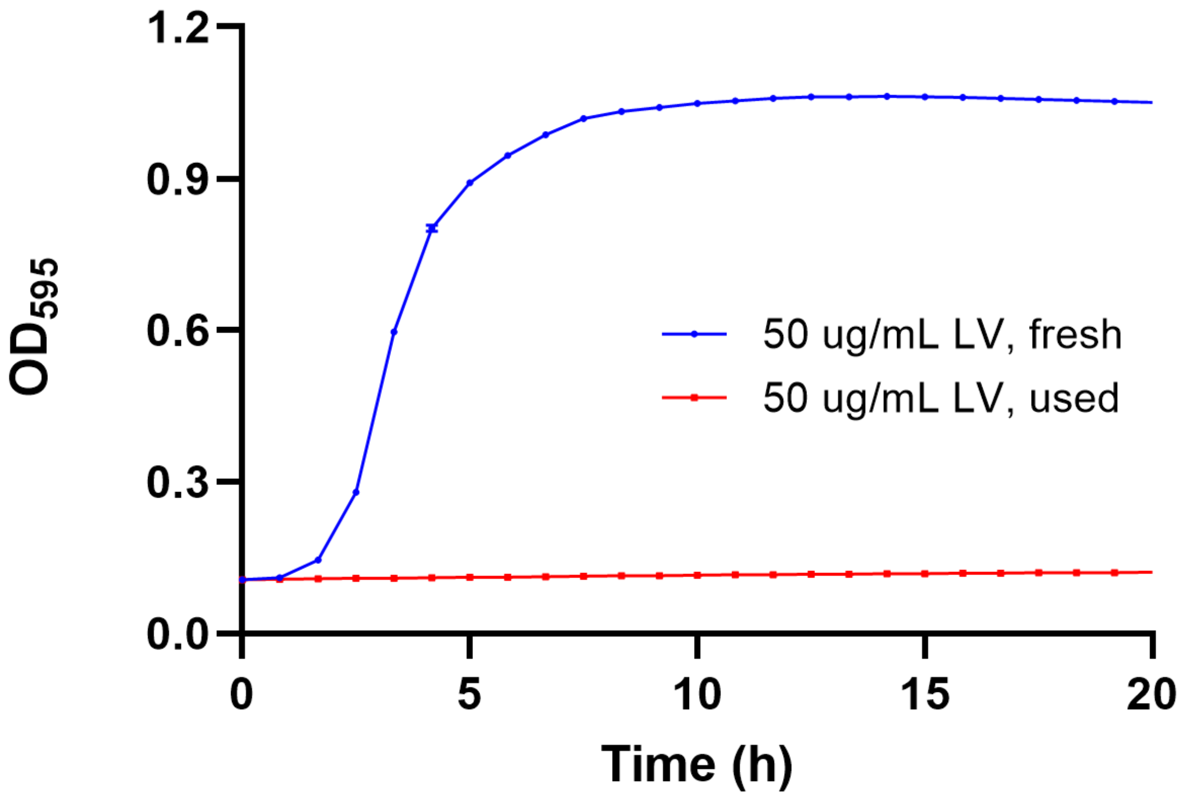

Figure S6 Media during the stationary phase co-culture survival assay is nutritionally exhausted. WT strain was grown in either fresh 50 ug/mL LV CDM or sterilely filtered used 50 ug/mL LV CDM from the 48-hour time-point. Data points and error represents the mean and SEM of six biologic replicates, respectively. For some data points, the error is smaller than the symbol used to plot the points.

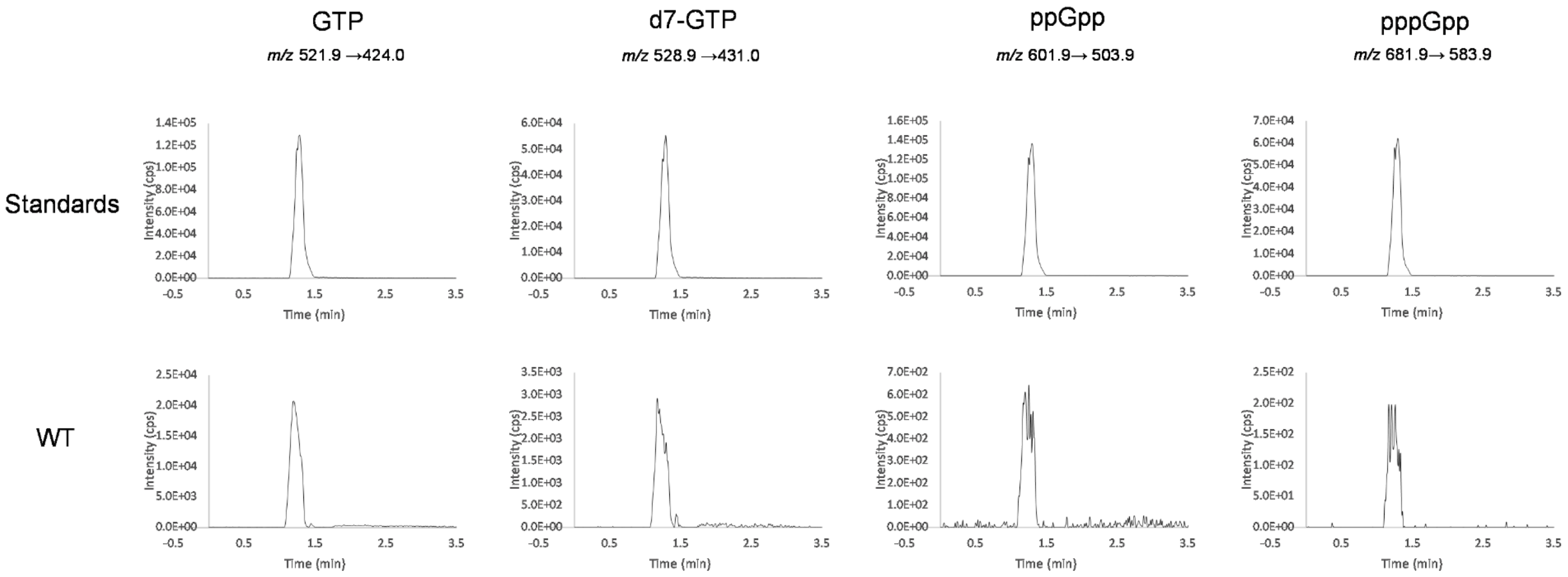

Figure S7 Extracted ion chromatograms for GTP, d7-GTP (internal standard), ppGpp, and pppGppp shown for analyte standards and the WT strain.

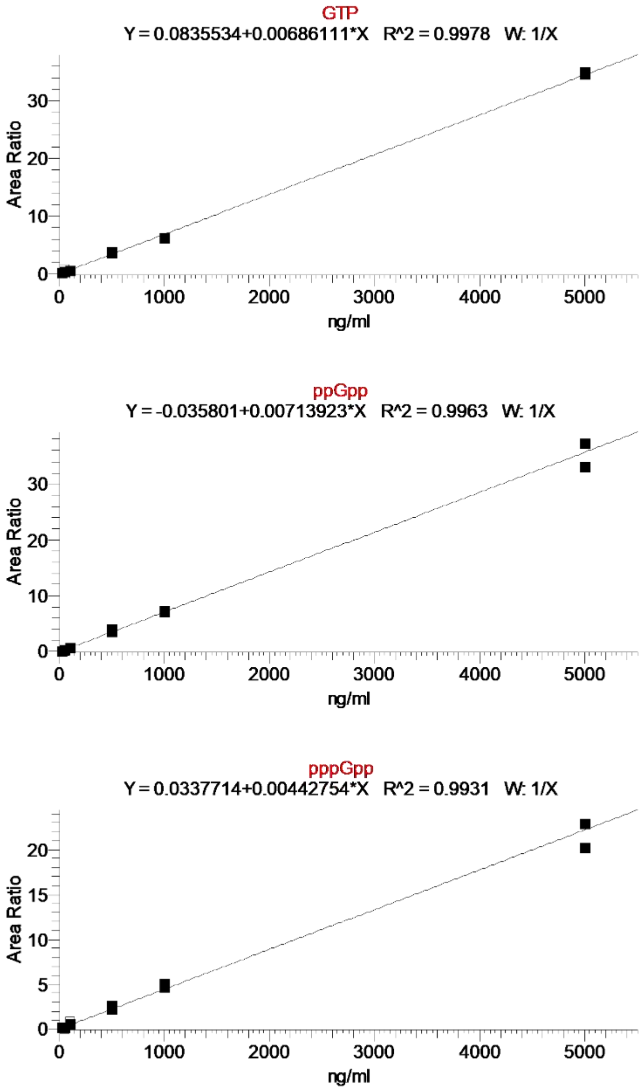

Figure S8 Standard curves produced by UPLC-MS/MS analyses for GTP, ppGpp, and pppGpp.
