# Supplementary material for: Clinical rel mutations in Staphylococcus aureus prime pathogen expansion under nutrient stress
Source: mSphere. 2023 Sep 26;8(5):e00249-23. doi: 10.1128/msphere.00249-23 (PMC10597345; doi:10.1128/msphere.00249-23)
Supplement: Supplemental Tables — Tables S1 to S6. [file msphere.00249-23-s0002.docx]

**Table S1** Clinical *rel* mutations.

| **Nucleotide Position** | **Mutation** | **Mutation Type** | **Amino Acid Positions** | **Protein Coding Change** | **Coding Change Type** |
| --- | --- | --- | --- | --- | --- |
| 400 | G>T | substitution | 134 | D>Y | substitution |
| 901 | G>A | substitution | 301 | A>T | substitution |
| 1150 | G>A | substitution | 384 | E>K | substitution |
| 2009 | T>G | substitution | 670 | V>G | substitution |
| 2089-2092 | ΔAATA | deletion | ≥697 | NISVM… > LVSW*… | frameshift, premature stop |

**Table S2** Statistical analysis of MIC data of strain panel. MIC values (μg/mL) were log2 transformed. Means and standard deviations (SD) are derived from N independent replicates. For each antibiotic, the mean value of each mutant strain was compared to the WT strain by Welch t-test. P values are adjusted for multiple comparisons. Statistical evaluation is not applicable (NA) when there is no difference in means and zero variance. MIC values for different strains were within a single 2-fold dilution for each antibiotic and there were no statistically significant differences (P < 0.05).

|  | **Vancomycin** | | | | **Daptomycin** | | | | **Ceftaroline** | | | | **Mupirocin** | | | |
| --- | --- | --- | --- | --- | --- | --- | --- | --- | --- | --- | --- | --- | --- | --- | --- | --- |
|  | **Mean** | **SD** | **N** | **P** | **Mean** | **SD** | **N** | **P** | **Mean** | **SD** | **N** | **P** | **Mean** | **SD** | **N** | **P** |
| WT | 1.00 | 0.00 | 7 | - | 0.71 | 0.49 | 7 | - | -2.00 | 0.00 | 7 | - | -2.00 | 0.00 | 5 | - |
| D134Y | 1.00 | 0.00 | 5 | NA | 0.60 | 0.55 | 5 | 0.72 | -2.00 | 0.00 | 5 | NA | -2.00 | 0.00 | 2 | NA |
| E384K | 1.00 | 0.00 | 6 | NA | 1.00 | 0.00 | 6 | 0.17 | -1.33 | 0.52 | 6 | 0.07 | -1.33 | 0.52 | 6 | 0.07 |
| V670G | 1.00 | 0.00 | 6 | NA | 0.67 | 0.52 | 6 | 0.87 | -2.00 | 0.00 | 6 | NA | -2.00 | 0.00 | 4 | NA |
| ΔACT | 1.00 | 0.00 | 4 | NA | 0.50 | 0.58 | 4 | 0.56 | -2.00 | 0.00 | 4 | NA | -2.00 | 0.00 | 2 | NA |

**Table S3** Raw unnormalized relative fitness values (*W*) of strains versus WT-GFP, in co-culture, in competitive growth fitness assays. Experiment 1 and Experiment 2 were performed on separate days with freshly made media.

| **Experiment 1** | | | | |
| --- | --- | --- | --- | --- |
| **50 ug/mL LV** | **WT** | **E384K** | **V670G** | **ΔACT** |
|  | 1.132665 | 0.922546 | 0.818827 | 0.676167 |
|  | 0.988917 | 0.921691 | 0.826217 | 0.695821 |
|  | 0.958651 | 0.88343 | 0.908926 | 0.686643 |
| **0.2 ug/mL LV** | **WT** | **E384K** | **V670G** | **ΔACT** |
|  | 1.099531 | 1.146667 | 1.163288 | 1.146852 |
|  | 1.040394 | 1.121031 | 1.197751 | 1.1732 |
|  | 1.041984 | 1.204478 | 1.216904 | 1.247581 |
| **Experiment 2** | | | | |
| **50 ug/mL LV** | **WT** | | **D134Y** | |
|  | 1.061135 | | 0.84747 | |
|  | 1.079821 | | 0.863759 | |
|  | 1.017631 | | 0.786435 | |
|  | 1.072455 | |  | |
|  | 1.029695 | |  | |
| **0.2 ug/mL LV** | **WT** | | **D134Y** | |
|  | 1.205826 | | 1.666144 | |
|  | 1.159515 | | 1.516287 | |
|  | 1.190786 | | 1.630477 | |
|  | 1.177306 | |  | |
|  | 1.147445 | |  | |

**Table S4** UPLC-MS/MS quantification of intracellular guanosine nucleotides during mid-exponential phase and represented as % composition of quantified guanosine nucleotide pool (GTP + ppGpp + pppGpp). In the comparison columns, mean values were compared using unpaired t-test. The (p)ppGpp^0^ (*Δrel ΔrelP ΔrelQ*) strain has no ability to synthesize (p)ppGpp in an RSH-dependent manner and serves as a control for baseline detection. Note that the LCMS signal peaks from the (p)ppGpp^0^ strain for ppGpp and pppGpp detection exhibited signal-to-noise ratios of >10:1 and were interpreted as true peaks, which may represent low-level detection of (p)ppGpp isomers and/or RSH-independent sources of (p)ppGpp. Subtracting this background signal does not alter the statistical analysis or interpretation.

| **Strain and Condition** | **% GTP** | | **% ppGpp** | | **% pppGpp** | | **Same strain**  **% (p)ppGpp**  **(± mupirocin)** | **Mutant vs. WT**  **% (p)ppGpp**  **(- mupirocin)** | **Mutant vs. WT**  **% (p)ppGpp**  **(+ mupirocin)** |
| --- | --- | --- | --- | --- | --- | --- | --- | --- | --- |
|  | **Mean** | **SEM** | **Mean** | **SEM** | **Mean** | **SEM** |  |  |  |
| WT | 64.6 | 1.0 | 19.9 | 1.2 | 15.5 | 0.2 | ****, P ≤ 0.0001 |  |  |
| WT + mupirocin | 34.3 | 1.6 | 37.4 | 4.1 | 28.3 | 2.5 |  |  |  |
| D134Y | 61.8 | 3.3 | 19.7 | 1.6 | 18.5 | 1.7 | **, P ≤ 0.01 | n.s. |  |
| D134Y + mupirocin | 38.2 | 1.1 | 33.0 | 2.0 | 28.8 | 1.0 |  |  | n.s. |
| E384K | 75.9 | 0.6 | 14.8 | 0.6 | 9.3 | 0.3 | ****, P ≤ 0.0001 | ***, P ≤ 0.001 |  |
| E384K + mupirocin | 26.9 | 0.7 | 47.3 | 3.7 | 25.8 | 3.1 |  |  | *, P ≤ 0.05 |
| V670G | 75.5 | 2.1 | 15.0 | 1.6 | 9.4 | 0.6 | ****, P ≤ 0.0001 | **, P ≤ 0.01 |  |
| V670G + mupirocin | 23.9 | 0.4 | 55.6 | 2.1 | 20.5 | 1.7 |  |  | **, P ≤ 0.01 |
| ΔACT | 76.1 | 1.3 | 14.3 | 1.1 | 9.6 | 0.3 | ****, P ≤ 0.0001 | **, P ≤ 0.01 |  |
| ΔACT + mupirocin | 38.1 | 0.6 | 35.4 | 0.8 | 26.6 | 0.7 |  |  | n.s. |
| (p)ppGpp^0^ | 86.1 | 2.1 | 7.1 | 1.1 | 6.8 | 1.0 |  | ***, P ≤ 0.001 |  |

**Table S5** Raw UPLC-MS/MS nucleotide concentrations of uninduced and mupirocin-induced strains during the mid-exponential phase.

| **Strain** | **GTP (ng/mL)** | **ppGpp (ng/mL)** | **pppGpp (ng/mL)** |
| --- | --- | --- | --- |
| WT 1 | 964.748 | 345.393 | 233.011 |
| WT 2 | 818.281 | 230.093 | 198.350 |
| WT 3 | 424.554 | 123.102 | 100.175 |
| WT induced 1 | 331.021 | 352.542 | 263.413 |
| WT induced 2 | 276.614 | 229.871 | 249.369 |
| WT induced 3 | 470.469 | 671.789 | 365.159 |
| D134Y 1 | 361.009 | 98.006 | 93.662 |
| D134Y 2 | 449.537 | 127.974 | 115.958 |
| D134Y 3 | 225.657 | 93.396 | 89.830 |
| D134Y induced 1 | 1278.230 | 1044.833 | 994.928 |
| D134Y induced 2 | 940.571 | 963.397 | 700.098 |
| D134Y induced 3 | 1207.135 | 920.945 | 887.043 |
| E384K 1 | 1490.204 | 318.289 | 186.929 |
| E384K 2 | 1588.362 | 290.183 | 202.923 |
| E384K 3 | 1266.822 | 238.929 | 144.157 |
| E384K induced 1 | 1204.499 | 1705.991 | 1363.911 |
| E384K induced 2 | 710.964 | 1368.945 | 573.098 |
| E384K induced 3 | 645.929 | 1258.610 | 596.073 |
| V670G 1 | 989.951 | 249.129 | 140.392 |
| V670G 2 | 1147.333 | 216.021 | 149.600 |
| V670G 3 | 1550.511 | 251.607 | 161.778 |
| V670G induced 1 | 983.127 | 2128.809 | 941.490 |
| V670G induced 2 | 603.287 | 1551.320 | 450.520 |
| V670G induced 3 | 754.547 | 1693.250 | 652.852 |
| ΔACT 1 | 1067.127 | 238.681 | 147.013 |
| ΔACT 2 | 1437.889 | 259.504 | 168.887 |
| ΔACT 3 | 772.382 | 124.100 | 96.089 |
| ΔACT induced 1 | 845.507 | 737.219 | 571.409 |
| ΔACT induced 2 | 744.811 | 734.642 | 505.212 |
| ΔACT induced 3 | 669.151 | 623.701 | 496.159 |
| (p)ppGpp^0^ 1 | 1405.662 | 157.148 | 149.936 |
| (p)ppGpp^0^ 2 | 1977.358 | 149.134 | 137.528 |
| (p)ppGpp^0^ 3 | 1844.191 | 116.554 | 113.550 |

**Table S6** Bacterial strains, plasmids, and primers.

| **Strain** | **Description** | **Source/Reference** |
| --- | --- | --- |
| Turbo | *E. coli* cloning strain | NEB |
| IM08B | *E. coli* strain containing methylase and specificity genes from *S. aureus* clonal complex 8 | Ref. 62 |
| JE2 | MRSA USA300 LAC cured of plasmids. | Dr. Christiane Wolz |
| WT-GFP | JE2 containing a chromosomally integrated and constitutively expressing GFP | This study |
| D134Y | JE2 containing a chromosomal Rel D134Y mutation | This study |
| E384K | JE2 containing a chromosomal Rel E384K mutation | This study |
| V670G | JE2 containing a chromosomal Rel V670G mutation | This study |
| ΔACT | JE2 containing a chromosomal Rel S645* mutation | This study |
| JE2_(p)ppGpp0_ | JE2 containing mutations in the synthetase domain of *relP*, *relQ*, and complete deletion of *rel*. | Ref. 60 |
| **Plasmid** | **Description** | **Source/Reference** |
| pTH100 | Plasmid for markerless genomic integration of GFP into *S. aureus* | Ref. 63 |
| pIMAY-Z | *S. aureus* allelic exchange vector | Ref. 61 |
| pMC101 | NEB HiFi DNA assembly of pIMAY-Z cut with EcoRI/BamHI and a PCR amplicon of wildtype genome using primers MC480/MC481 | This study |
| pMC102 | NEB HiFi DNA assembly of pIMAY-Z cut with EcoRI/BamHI and a PCR amplicon of clinical strain containing Rel V670G mutation using primers MC480/MC481 | This study |
| pMC105 | NEB HiFi DNA assembly of pIMAY-Z cut with EcoRI/BamHI and a PCR amplicon of clinical strain containing Rel E384K mutation using primers MC484/MC485 | This study |
| SA-51 | NEB Q5 site directed mutagenesis of plasmid pMC101 using primers EC134/EC135 | This study |
| SA-53 | NEB HiFi DNA assembly of pIMAY-Z cut with EcoRI and a PCR amplicon of clinical strain containing Rel D134Y mutation using primers EC136/EC137 | This study |
| **Primer** | **Description** | **Source/Reference** |
| MC480 | tatcgataagcttgatatcgtccaaattcacctgttttc | This study |
| MC481 | cggccgctctagaactagtgaaatcgtctagtgccaaag | This study |
| MC484 | tatcgataagcttgatatcgcgtaacttcttgtgcttc | This study |
| MC485 | cggccgctctagaactagtgcggctcttcgttatattg | This study |
| EC134 | tgggtaaaataaaaagacgcaac | This study |
| EC135 | ttcaacattaattagtcgttc | This study |
| EC136 | tatcgataagcttgatatcgttcagcttcaaccatcattc | This study |
| EC137 | tggatcccccgggctgcagggaaacagtagatttaggtcg | This study |
